# Prognostic Factors in Cutaneous Squamous Cell Carcinoma: Is Patient Delay in Hospital Visit a Predictor of Survival?

**DOI:** 10.5402/2011/285289

**Published:** 2011-11-14

**Authors:** Y. Endo, M. Tanioka, Y. Miyachi

**Affiliations:** Department of Dermatology, Graduate School of Medicine, Kyoto University, 54 Kawahara-cho, Shogoin, Sakyo-ku, Kyoto 606-8507, Japan

## Abstract

The patient's delay in the visit to a hospital seems to play an important role in prognosis in invasive cutaneous squamous cell carcinoma (SCC). This report explored prognostic factors of cutaneous SCC focusing on patient delay in hospital visit. Data of 117 Japanese patients who were treated for invasive cutaneous SCC in our facility between 2000 and 2010 were used for analysis. A multivariate Cox proportional-hazard modelling revealed that a pair of TNM stage (hazard ratio, 5.0; 95% CI, 1.8 to 13.9) and poorer histological differentiation (hazard ratio, 3.2; 95% CI, 0.93 to 10.3), and a pair of tumour size (hazard ratio, 1.02; 95% CI, 1.004 to 1.04) and rapid growth (hazard ratio, 8.25; 95% CI, 1.29 to 52.7) were a prognostic factor whereas patient delay in hospital visit was not. However, patient delay in hospital visit was correlated with larger tumour size.

## 1. Introduction

Cutaneous squamous cell carcinoma (SCC) is a malignant tumour of keratinocytes that tends to metastasise and leads to mortality. In cutaneous SCC, a number of clinical and histological factors have been found to be poor prognostic factors. For example, tumour size, gender, preceding lesion, histological findings such as degree of the differentiation, and tumours location have been reported as a prognostic factor of local recurrence, metastasis, and disease-specific death [2–5]. In addition, recent research has pointed out that delay before surgical removal is an independent prognostic factor because delay may lead to more advanced stage [4]. In this observational study, we attempted to explore prognostic factors of cutaneous SCC, especially paying attention to the effect of patient delay in hospital visit. We hypothesised the structure that prolonged patient's delay in the visit to a hospital affects stage and prognosis consequently. 

## 2. Patients and Methods

A patient registry system from the Department of Dermatology at the Kyoto University Hospital was accessed to retrieve medical information on patients in whom invasive SCC of the skin was histopathologically diagnosed and treated from 2000 to 2010. During that period, one hundred and forty-nine patients were retrieved. Twenty cases were excluded because of tumour in situ, and two cases because of lack in clinical and pathological information. Ten cases with genital SCC were excluded because of different staging system. The remaining 117 Japanese patients who were treated for invasive cutaneous SCC were eligible for analysis. Followup was performed every 3 to 6 months by physical examination and blood works. A mean followup was 29.9 months. Variables were demographic characteristics, TNM stage of cutaneous squamous cancer (UICC 7th edition, 2009) [1], tumour site, histopathological findings (differentiation of the tumour, perineural invasion, and lymphovascular invasion), rapid growth [6], radiation therapy, and preceding condition of tumour site. Degree of the differentiation was rated on an ordinal scale of poor, moderate, or well differentiated. Perineural invasion, lymphovascular invasion, rapid growth, radiation therapy, and preceding condition of tumour site were collapsed into dichotomous variables, with 0 as indicative of negative and 1 as indicative of positive. Patient delay in hospital visit was defined as time taken before hospital visit with complaint of SCC since the patient had noticed the lesion. Primary endpoint was set as disease-specific death. 

### 2.1. Statistical Analysis

Descriptive statistics were calculated to estimate the frequencies, means, and standard deviations of the study variables. Survival analyses were conducted from the date when a patient had noticed the tumour to the date of death with use of the Kaplan-Meier method. Data from patients who were alive at the study followup (31, December, 2010) were censored on that date. 

Preliminary analysis was conducted exploring whether patient delay in hospital visit varies according to change of other prognostic factors. As distribution of patient delay in hospital visit was skewed right, Spearman rank correlation coefficients and Mann-Whitney *U* test were used. 

Then, a Cox proportional-hazards model was used for univariate and multivariate analysis to evaluate the effect of predictors on disease-specific survival [7]. First, the significant predictors on the survival were screened via univariate analysis. Second, variables that had significant contribution on survival in univariate analysis were entered into the multivariate model, followed by a backward elimination procedure that was used with *P* based on Wald statistics higher than 0.1 for variable removal from the model. As a pair of stage and tumour size showed multicollinearity, either of them was used for the initial multivariate model. The results were adjusted for age. The SPSS 12.0 (SPSS Inc., Chicago, Ill, USA) was used for all statistical analyses. Missing values were excluded from each analysis. All given probabilities are two tailed and the significance levels were set at 5%. 

## 3. Results

A total of 117 patients were included into the analysis. The characteristics of the patients were shown in Table 1. The mean age of patients was 74.4 ± 12.7 years (range, 30 to 98 years); 74 (63%) were male. SCC occurred frequently in the cheek (21%), lower extremities (16%), and ear (8.5%). Actinic keratosis (25%) was most frequent preceding condition, followed by Bowen disease (15%), both of which are associated with sun exposure (Table 1). Patient delay was 2.97 ± 4.1 years and did not significantly differ amongst locations of tumour. Of 117 patients, there were 8 deaths (6.8%) recorded as caused by squamous cell carcinoma during the follow-up period. The 5-year survival rate was 87% in total. One hundred percent for stage I, 82% for stages II, 83% for stage III. All cases of stage IV were dead or censored within 6 months. Survival curves of Stage II and III did not differ significantly (Log rank statistics 0.29, *P* = 0.59). 

Preliminary analysis revealed that patient delay in hospital visit was associated with other prognostic determinants. Longer patient delay was significantly correlated with larger tumour size (Spearman rho rank correlation coefficient = 0.20, *P* = 0.04) and less rapid growth (Mann-Whitney *U* = 50, *P* = 0.049). 

Of 117, eighty-eight cases were considered in the Cox regression analysis, as 18 cases had missing values, and 11 cases were censored before the earliest event occurred. In univariate analysis tumour size, TNM stage, rapid growth, lymphovascular invasion, and the differentiation were identified as a significant determinant, while patient delay in hospital visit, age, and sex were not. Multivariate model revealed that a pair of TNM stage and poorer histological differentiation and a pair of tumour size and rapid growth were a prognostic factor (Table 2). 

## 4. Discussion

The current study addressed the question whether patient delay in hospital visit and other factors affect the prognosis of invasive cutaneous SCC using multivariate analysis. As expected, tumour size, TNM stage, differentiation of the tumour [2, 3], and rapid growth were thought to be a prognostic factor, while patient delay in hospital visit was not. 

Based on the literature [4], we first hypothesized that patients with longer patient delay in hospital visit had been prone to advanced disease and poor prognosis. In this study, correlation analysis revealed only the weak tendency that prolonged delay was associated with slower tumour growth and larger tumour size that is an important prognostic factor of cutaneous SCC. In other words, patients tend to postpone their visit to a hospital only in case the tumour grows slowly, which leads to larger tumour as a result. This tendency was confirmed by the fact that the significance of the correlation between patient delay in hospital visit and tumour size was maintained at a slightly elevated level when speed of tumour growth was partialed out. Although patient delay in hospital visit was not directly associated with prognosis, it can be said that patient delay may indirectly affect prognosis of cutaneous SCC. Further public announcement seems important to encourage people to consult a dermatologist when they notice skin tumours so as to shorten the delay in hospital visit. 

In contrast, the differentiation of tumour was shown to be an independent prognostic factor as has been expected. As differentiation of tumour is not considered when staging, careful followup is required for cases with cutaneous SCC of poor differentiation irrespective of stage. 

In this study, patients with genital SCCs were not considered because of different staging system and small sample size. Genital SCCs tended to be at more advanced stage and of poorer differentiation than SCCs in other site at the time of diagnosis [8]. This phenomenon may be explained by human papilloma virus (HPV) infection because community-based epidemiological study revealed that HPV infection in genital area is associated with cancer-related death [9]. HPV infection may accelerate tumour progress of cutaneous SCC. 

Several limitations of the study deserve mention. First, this study was conducted at a single medical facility and consisted of a relatively smaller sample, thereby limiting generalization of the results to patients of other hospitals. In addition, because the sample lacked diversity with respect to race, we were unable to assess the effect of that important factor on outcomes. However, our study reflects the prognostic factors of cutaneous SCC in Japanese population. 

In summary, our study showed that tumour size, TNM stage, differentiation of the tumour, and rapid growth were thought to be a prognostic factor, while prolonged patient delay seems to affect indirectly the prognosis by influencing larger tumour size.

## Figures and Tables

**Table 1:** Characteristics of the patients, given as mean ± standard deviation or no, (%) (*N* = 117).

Prognostic factor	Number (%)
Sex, male (%)	74 (63)
Age (year)	74.4 ± 12.7
Patient delay in hospital visit (year)	2.97 ± 4.1
Tumour size (mm)	28.3 ± 28.7
Lymph node metastasis	12 (10.3)
TNM stage^ (1)^	
Stage I	56 (47.9)
Stage II	38 (32.5)
Stage III	17 (14.5)
Stage IV	6 (5.1)
Radiation related	3 (2.6)
Rapid growth	7 (6.0)
Perineural invasion	3 (2.6)
Lymphovascular invasion	3 (2.6)
Degree of differentiation	
Well	67 (57.3)
Moderate	27 (23.1)
Poor	7 (6.0)
Tumour site	
Temporal head	8 (6.8)
Frontal head	4 (3.4)
Eye lid	1 (0.9)
Cheek	24 (20.5)
Ear	10 (8.5)
Nose	4 (3.4)
Lip	5 (4.3)
Upper extremities	5 (4.3)
Finger	8 (6.8)
Hand	8 (6.8)
Trunk	6 (5.1)
Lower extremities	19 (16.2)
Planter of the foot	3 (2.6)
Dorsum of the foot	3 (2.6)
Heel	2 (1.7)
Digit of the foot	7 (6.0)
Preceding lesion	
Actinic keratosis	29 (24.8)
Basal cell carcinoma	1 (0.9)
Bowen disease	17 (14.5)
Burn	3 (2.6)
Epidermal cyst	1 (0.9)
Keratoacanthoma	6 (5.1)
Pustulosis palmaris and plantaris	1 (0.9)
Radiation keratosis	1 (0.9)
Scar	4 (3.4)
Other squamous cell carcinoma	1 (0.9)
Trichilemmal cyst	1 (0.9)
Ulcer	10 (8.5)
Unknown	42 (35.9)

Note: ^(1)^Stage was described using UICC (7th edition, 2009) [1].

**Table 2:** Prognostic factors of cutaneous squamous cell carcinomas influencing overall survival: results of univariate and multivariate Cox proportional hazard models.

Prognostic factor	Univariate model			Multivariate model 1^(1)^			Multivariate model 2^(1)^		
	HR	95% CI	*P*	HR	95% CI	*P*	HR	95% CI	*P*
Age	1.02	0.96–1.08	0.57	1.02	0.94–1.1	0.58	1.04	0.97–1.12	0.27
Patient delay in hospital visit	0.97	0.79–1.19	0.78						
Sun exposure (positive = 1)	1.83	0.37–9.10	0.46						
Tumour size	1.02	1.01–1.04	0.01				1.02	1.004–1.04	0.01
Lymph node metastasis (positive = 1)	3.03	0.61–15.1	0.18						
TNM Stage^(2)^	4.02	1.60–10.1	0.04	5.02	1.79–14.1	0.00			
Radiation related (true = 1)	4.90	0.63–40.2	0.14						
Rapid growth (positive = 1)	7.60	1.49–39.1	0.02				8.25	1.29–52.7	0.03
Perineural invasion (positive = 1)	4.50	0.55–36.4	0.16						
Lymphovascular invasion (positive = 1)	8.15	0.942–70.6	0.06						
Degree of differentiation	2.99	1.27–7.64	0.02	3.20	0.94–10.9	0.06			

Note: The total number of cases was 117; of those, 88 were considered in the analysis, as 18 cases had missing values, and 11 cases were censored before the earliest event occurred. There were 8 deaths recorded as caused by squamous cell carcinoma.

^(1)^Variables that were significant in univariate analysis were entered into the model, followed by a backward elimination procedure with *P* > 0.1 for variable removal from the model. In model 3, tumour size was used instead of TNM stage. The results were adjusted for age. CI: confidence interval; HR: hazard ratio.

^(2)^Stage was described using UICC (7th edition, 2009).
